# Complication Spectrum Following Expanded Endoscopic Endonasal Surgery for Intradural Skull Base Tumors: A Systematic Review and Meta-Analysis

**DOI:** 10.3390/brainsci16080887

**Published:** 2026-08-20

**Authors:** Badr Hafiz, Thamer Alsharif, Faisal Sukkar, Fahad Okal, Maryam Enani, Moaath Alghamdi, Abdulrazag Ajlan, Mohammed Aref, Mohammed Binmahfoodh, Saleh Baeesa

**Affiliations:** 1 Department of Neurosciences, King Faisal Specialist Hospital and Research Centre, Prince Saud Al-Faisal Road, P.O. Box 40047, Jeddah 21499, Saudi Arabia; neurosurg.badr@gmail.com (B.H.); maref@kfshrc.edu.sa (M.A.); binmahfoodh@kfshrc.edu.sa (M.B.); 2 Neurosurgery Department, King Abdulaziz Specialist Hospital, Taif 26521, Saudi Arabia; thameralsharif@outlook.com (T.A.); moaath.gh7@gmail.com (M.A.); 3 Department of Neurosurgery, Dr. Sulaiman Al Habib Medical Group Holding Company, Jeddah 23618, Saudi Arabia; sukkarfaisal@gmail.com (F.S.); dr_ajlan79@hotmail.com (A.A.); 4 Department of Neurosurgery, King Abdulaziz Medical City, National Guard Health Affairs, Jeddah 22384, Saudi Arabia; dr.3okal@gmail.com; 5 Neurosurgery Section, Department of Surgery, King Abdulaziz University Hospital, Jeddah 22252, Saudi Arabia; mariamenani1995@gmail.com; 6 Faculty of Medicine, Alfaisal University, Riyadh 11533, Saudi Arabia

**Keywords:** expanded endoscopic endonasal approach, skull base tumors, intradural tumors, postoperative complications, cerebrospinal fluid leak, meningitis, neurological deficit, vascular injury, seizures

## Abstract

**Highlights:**

**What are the main findings?**

- CSF leak was the most consistently reported complication after expanded endoscopic endonasal surgery for adult intradural skull base tumors, with a strict adult-only random-effects incidence of 23.0%.
- Meningitis, neurological/vascular complications, and postoperative seizures were uncommon and could only be summarized descriptively because of sparse evidence.

**What are the implications of the main findings?**

- Standardized complication reporting is needed to improve future evidence synthesis and comparisons across institutions.
- Large mixed endoscopic endonasal cohorts should not replace pathology-specific adult intradural outcome reporting.

**Abstract:**

**Background/Objectives:** Expanded endoscopic endonasal approaches (EEAs) provide direct ventral access to adult intradural skull base tumors; however, postoperative complications are variably defined and reported in the literature. This review aimed to synthesize the overall spectrum of adult intradural EEA complications and distinguish pathology-specific evidence from large mixed EEA complication cohorts. **Methods:** A PRISMA 2020 systematic review and meta-analysis searched PubMed/MEDLINE, Embase, and Web of Science from database inception through July 2026. Eligible studies included adults or adult-separable subgroups undergoing expanded EEAs for intradural skull base tumors. The primary outcomes were CSF leak, meningitis or intracranial infection, new neurological deficit or vascular injury/stroke, hydrocephalus, mortality, and postoperative seizures. Pathology-specific adult intradural series with exact counts were quantitatively synthesized when appropriate, and large mixed EEA cohorts were summarized separately when the eligible subgroup was not separable. **Results:** After full-text eligibility assessment, 48 reports were retained in the extraction-status set. Three strictly adult-only exact-count reports contributed to the primary CSF leak meta-analysis, yielding a random-effects incidence of 23.0% (95% CI, 10.5–43.1%; I^2^ = 78.7%). A sensitivity analysis adding one mixed-age cohort yielded 23.8% (95% CI, 14.5–36.6%; I^2^ = 68.9%), while a broader sensitivity analysis including both mixed-age reports yielded 21.1% (95% CI, 12.2–33.8%; I^2^ = 69.4%). Meningitis was reported in 6/166 patients across four studies (3.6%; 95% exact CI, 1.3–7.7%), and new neurological deficit/vascular injury/stroke was reported in 4/166 patients across four studies (2.4%; 95% exact CI, 0.7–6.1%). Postoperative seizures were reported in 4/652 patients across two studies (0.6%; 95% exact CI, 0.2–1.6%). Large mixed EEA cohorts were summarized separately because adult intradural tumor subsets could not be separated. **Conclusions:** CSF leak was the most consistently reported complication after expanded EEAs for adult intradural skull base tumors. The strict adult-only random-effects estimate was 23.0%, but it was based on only three studies and showed substantial heterogeneity, limiting its generalizability. Meningitis, neurological/vascular events, and seizures were uncommon in the extractable data and were summarized descriptively rather than being meta-analyzed. Large mixed EEA cohorts provide important safety context but cannot substitute for standardized adult intradural, pathology-specific reporting.

## 1. Introduction

Intradural skull base tumors encompass a diverse group of neoplasms. Meningiomas are the most prevalent of these tumors, representing 37.6% of all primary central nervous system (CNS) tumors with an overall incidence of 8.83 per 100,000 population; approximately one-third involve the skull base [1]. Rarer midline pathologies, including craniopharyngiomas and chordomas, occur at rates of 0.19 and 0.08 per 100,000, respectively, yet their deep-seated locations continue to challenge traditional surgical corridors [1].

Over the past two decades, expanded endoscopic endonasal approaches (EEAs) have gained widespread adoption because they provide direct ventral access to these lesions, eliminate the need for brain retraction, and reduce the manipulation of critical neurovascular structures [2,3]. Despite these advantages, EEAs carry a meaningful complication burden. A systematic review of endoscopic endonasal anterior skull base surgery reported a major complication rate of 8.8%, but adult intradural tumor-specific estimates for CSF leak, meningitis, neurovascular events and seizures remain unevenly reported [4].

Postoperative complications after intradural EEAs include CSF leakage, meningitis, vascular injury, new neurological deficits, hydrocephalus, endocrinological morbidity, and seizures. CSF leaks are particularly relevant because expanded intradural corridors create large dural defects and high-flow leaks, whereas neurological and infectious events directly influence recovery, readmission risk, and long-term function. Seizures remain clinically important within this complication spectrum, particularly in the context of intracranial and skull base tumor surgery, but they should be interpreted as one neurological adverse event rather than as the sole determinant of postoperative safety [4,5,6,7].Existing reviews have addressed either broad anterior skull base complications or individual outcomes but have not provided a transparent synthesis of the adult intradural complication spectrum that distinguishes pathology-specific series from large mixed EEA cohorts. Borg et al. [4] summarized anterior skull base surgical complications, whereas Lai et al. [8] examined postoperative seizures. Both provide useful context, but adult-only intradural data, exact outcome counts, and reconstruction-related complications remain inconsistently separable in the literature.

More recent comparative evidence has also evaluated seizure risk after open and expanded endoscopic endonasal approaches for intradural skull base tumors [9]. Although routine anticonvulsant prophylaxis is not generally supported in seizure-naïve patients with newly diagnosed brain tumors [10], seizure risk remains clinically relevant in the setting of skull base lesions and their surgical treatment [11].

This systematic review and meta-analysis aimed to synthesize the spectrum of postoperative complications following expanded EEAs for adult intradural skull base tumors. The primary outcomes were CSF leakage, meningitis or intracranial infection, new neurological deficit or vascular injury/stroke, hydrocephalus, mortality, and postoperative seizures. Quantitative pooling was restricted to clinically defensible exact-count subsets, whereas large mixed EEA cohorts were retained as contextual evidence.

## 2. Methods

### 2.1. Study Design

This systematic review and meta-analysis evaluated postoperative complications following expanded EEAs for adults with intradural skull base tumors. This review followed the Preferred Reporting Items for Systematic Reviews and Meta-Analyses (PRISMA) 2020 reporting standards [12] and Cochrane Handbook principles [13], including duplicate screening and extraction, explicit handling of missing data, and quantitative synthesis only when outcome definitions and numerators/denominators were sufficiently comparable. The protocol was registered with PROSPERO (CRD420261427805; registration date: 25 June 2026), and institutional review board approval was not required.

The review question was framed using the Population, Intervention, Comparator and Outcome (PICO) format. The population comprised adults aged ≥18 years with intradural skull base tumors; the intervention was expanded EEAs or equivalent extended endoscopic endonasal skull base surgery; and the comparator, when available, was transcranial or open skull base surgery. Outcomes included the postoperative complication spectrum, including CSF leak, meningitis or intracranial infection, hemorrhage, vascular injury or stroke, new neurological deficit, hydrocephalus, mortality, length of stay, recurrence/progression, and postoperative seizures.

### 2.2. Eligibility Criteria

Studies were eligible if they met all of the following criteria: human clinical study; adult patients or an adult-separable subgroup; expanded endoscopic endonasal surgery for intradural skull base tumor pathology; peer-reviewed full-text English-language publication; randomized trial, prospective or retrospective cohort, comparative study, or case series with at least five patients; and extractable postoperative complication data.

Studies were excluded if they were pediatric-only cohorts, mixed pediatric/adult cohorts without adult-separable outcome data, purely extradural lesion series, standard pituitary adenoma series without intradural extension, non-tumor studies, case reports or series with fewer than five patients, conference abstracts, editorials, letters, reviews without independent original data, technical notes without extractable outcomes, animal or cadaveric studies, duplicate cohorts without separable non-overlapping data, or reports lacking postoperative complication outcomes. Mixed pediatric/adult reports without adult-separable outcomes were not eligible for strict adult-only primary synthesis. Otherwise, relevant mixed-age reports with exact whole-cohort outcome counts were retained only as explicitly labeled sensitivity/contextual evidence.

Eligible tumor types included meningioma, craniopharyngioma, chordoma, or chondrosarcoma with intradural extension or eligible expanded skull base involvement; epidermoid or dermoid tumors; schwannoma; Rathke cleft cyst with intradural/suprasellar extension; and other intradural skull base tumors. Chordoma, chondrosarcoma, clival tumors, Rathke cleft cysts, and mixed skull base series were screened carefully because eligibility depended on whether adult intradural tumor data and complication data were extractable.

### 2.3. Search Strategy

The search strategy included PubMed/MEDLINE, Embase, and Web of Science. Databases were searched from inception to July 2026. Backward citation tracking of relevant systematic reviews and included studies was performed to identify additional reports.

The core search combined endonasal approach terms, intradural skull base tumor terms, and broad complication terms: (“endoscopic endonasal” OR “expanded endoscopic endonasal” OR “extended endonasal” OR EEA OR “endoscopic skull base”) AND (intradural OR “skull base tumor” OR meningioma OR craniopharyngioma OR chordoma OR chondrosarcoma OR epidermoid OR dermoid OR schwannoma OR “Rathke cleft cyst”) AND (complication OR “cerebrospinal fluid leak” OR CSF OR meningitis OR infection OR hemorrhage OR vascular OR stroke OR “neurological deficit” OR hydrocephalus OR mortality OR seizure). A supplementary seizure-specific term set was retained only to improve sensitivity for that complication and did not define eligibility or the review objectives.

### 2.4. Study Selection

Two reviewers (BH and TA) independently screened the study titles and abstracts. Potentially relevant records underwent full-text review. Disagreements were resolved by consensus, and a principal investigator (SB) was consulted for unresolved disagreements. Duplicate cohorts were handled by retaining the most complete, pathology-specific, or most recent report unless clearly non-overlapping outcome data were extracted from multiple publications.

For publications containing both EEA and transcranial cohorts, EEA data were extracted separately, and comparative estimates were extracted only when numerators and denominators were available for both groups. Record-level identification, deduplication, and exclusion counts were documented only when supported by retained search documentation and were not reconstructed from the incomplete records. Accordingly, the PRISMA diagram reports only verifiable aggregate counts of the included studies. The 48 reports constituted the full-text evidence set carried into outcome-specific extraction/status assessment; quantitative denominators varied by endpoint because only reports with exact, clinically interpretable adult intradural numerators and denominators contributed to the primary synthesis.

### 2.5. Data Extraction

Two reviewers independently extracted the study-level data using a standardized form. The variables included author, year, study design, recruitment period, tumor pathology and location, number of EEA patients, comparator size, adult-only status, intradural eligibility, reconstruction method when reported, follow-up, CSF leak, meningitis or intracranial infection, hemorrhage, stroke or vascular injury, new neurological deficit, hydrocephalus, mortality, length of stay, recurrence/progression, postoperative seizures, and antiseizure medication use.

For all outcomes, numerators and denominators were extracted directly whenever possible. Percentages were converted to counts only when the denominator made the conversion exact, and the resulting count was explicitly verifiable. Rounded or subgroup-ambiguous percentages were described but not pooled. Supplementary Table S1 reports the outcome-specific extraction status for all 48 reports retained after full-text eligibility assessment and provides the reason when an adult intradural count was not quantitatively usable. For the reviewer-requested study characteristics’ summary, original publications were rechecked for country/institution, age composition, EEA terminology, and source-level complication descriptions; fields that remained unverifiable were not inferred.

### 2.6. Outcomes

The outcomes included the full spectrum of postoperative complications after expanded EEAs. A postoperative CSF leak was synthesized only when an exact postoperative numerator and denominator could be extracted. Reports that did not clearly separate postoperative from intraoperative leakage or provided only rounded or subgroup-ambiguous percentages were not included in the strict adult-only primary meta-analysis. Meningitis/intracranial infection and neurological/vascular outcomes were retained according to source-report definitions and summarized descriptively because the data contained zero-event studies, few total events, and non-identical definitions. A definition-restricted sensitivity meta-analysis was not performed because too few studies used sufficiently uniform definitions of the outcomes. Postoperative seizures were retained as neurological complications and summarized descriptively.

### 2.7. Risk of Bias Assessment

Comparative nonrandomized studies were assessed using the Risk of Bias in Non-randomized Studies of Interventions (ROBINS-I) tool. Noncomparative case series contributing extractable quantitative outcomes were assessed using the Joanna Briggs Institute (JBI) Critical Appraisal Checklist for Case Series. The risk-of-bias domains included patient selection, ascertainment of exposure/intervention, outcome ascertainment, completeness of follow-up, adequacy of reporting, confounding, and selective reporting. Studies screened but not contributing exact quantitative outcomes were summarized in the extraction-status table and were not assigned per-study risk-of-bias judgments.

Studies that contributed to exact quantitative outcomes underwent a study-level risk-of-bias assessment. Reports without extractable adult intradural outcome counts were summarized in the extraction-status table and were not included in the per-study risk-of-bias judgments.

### 2.8. Structured Qualitative Certainty Assessment

Outcome-level certainty was summarized using a structured qualitative framework based on domains commonly considered in certainty assessment: study limitations/risk of bias, inconsistency, indirectness, imprecision, and potential publication bias. This was not presented as a formal GRADE assessment because the evidence base consisted predominantly of observational case series and non-randomized cohorts, and because outcome-level data were sparse. Therefore, the judgments were descriptive and conservative. Publication bias for the primary CSF leak meta-analysis was not formally assessed using funnel-plot asymmetry or Egger’s regression because only three strictly adult-only studies contributed; with so few studies, such tests would be severely underpowered and potentially misleading.

### 2.9. Statistical Analysis

Analyses were reproduced in Python 3.13.5 using StatsModels 0.14.6 and SciPy 1.17.0. For the primary CSF leak analysis, only strictly adult-only exact-count proportions were logit-transformed and combined using inverse-variance random-effects meta-analysis with the DerSimonian–Laird estimator for between-study variance. The works of Koutourousiou et al., 2013 [14] (47 adults and 17 children; overall CSF leak 15/64) and Liu et al., 2023 [15] (age range 7–61 years; CSF leak 1/21) were excluded from the primary analysis because exact adult-specific CSF leak numerators were not independently extractable. The work of Koutourousiou et al., 2013 [14] was added in a first sensitivity/contextual analysis, and that of Liu et al., 2023 [15] was added in a broader second sensitivity/contextual analysis. Individual study confidence intervals and descriptive rare-event confidence intervals were calculated using the Clopper–Pearson exact method. No meta-analytic model was fitted for meningitis, neurological/vascular events, or seizures; events and denominators were summed descriptively, and exact binomial confidence intervals were reported. No continuity correction was applied.

Heterogeneity was assessed using Cochran’s chi-square test and I^2^. I^2^ values were interpreted cautiously because the estimates may be imprecise when few studies are included. Values of 0–40% may not be important, 30–60% may represent moderate heterogeneity, 50–90% substantial heterogeneity, and 75–100% considerable heterogeneity. For outcomes represented by a single eligible study, heterogeneity statistics were not estimated in this study. Funnel plot asymmetry and Egger’s regression were not performed because the primary CSF leak analysis contained only three studies; such tests are severely underpowered with fewer than 10 studies.

For comparative EEA versus transcranial/open cohorts, odds ratios (ORs) with 95% CIs were extracted or calculated when both numerators and denominators were available. Subgroup and sensitivity analyses were performed considering pathology, skull base region, mixed-age status, unclear intradural eligibility, duplicate-risk institutional cohorts, and rounded-percentage reporting. Large mixed EEA complication cohorts were summarized separately when adult intradural tumor subgroups were not separable.

A publication-era comparison was retained only as a non-inferential exploratory description. Because the available reports were pathology-mismatched, center-specific, and used publication year as a proxy for operative era, a comparison was not used in the abstract, conclusion, or causal interpretation.

## 3. Results

### 3.1. Study Selection

After the full-text eligibility assessment, 48 reports were retained in the extraction-status set after the removal of one citation outside the revised search period. These reports were carried forward for outcome-specific extraction and status assessment and were organized into pathology-specific adult intradural evidence and large mixed EEA complication cohorts. Retention in this set did not mean that every report contributed to a quantitative or descriptive end-point. Only outcome-specific subsets with exact, verifiable counts contributed to the estimates; therefore, the number of contributing studies and patients differed across endpoints.

**Figure 1:**
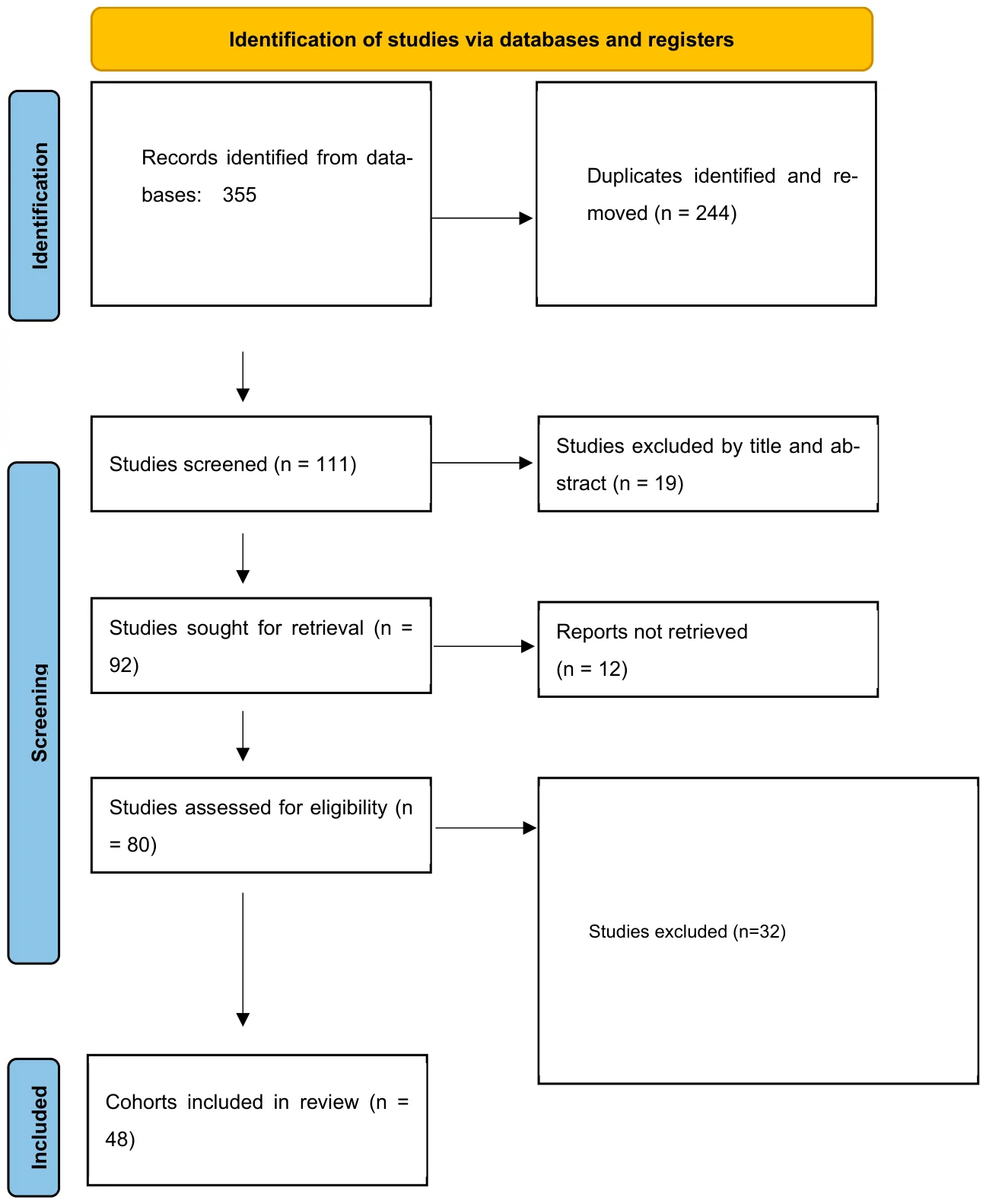
PRISMA flow diagram.

A complete record-level search log was not found. The retained documentation supports the aggregate PRISMA counts shown in Figure 1 (355 records identified, 244 duplicates removed, 111 records screened, 92 reports sought for retrieval, 80 reports assessed for eligibility, 32 reports excluded, and 48 reports retained); however, it does not support a reliable category-by-category reconstruction of the reasons for all 32 full-text exclusions. Therefore, we did not retrospectively reconstruct exclusion categories without supporting documentation. This limitation is explicitly acknowledged. The quantitative synthesis was limited to exact, verifiable, and clinically comparable data. Supplementary Table S1 presents the outcome-specific extraction status of the 48 retained reports.

### 3.2. Study Characteristics

The 48 reports retained after the full-text eligibility assessment are summarized in Table 1. Most were retrospective single-center case series or nonrandomized comparative cohorts published between 2006 and 2024. Importantly, the retained set included both pathology-specific intradural series and large mixed EEA complication cohorts, which were analyzed in separate tiers because the latter commonly included pituitary, extradural, pediatric, or procedure-level data that could not be separated for the target population.

Table 1 provides a concise overview of all 48 retained reports and their evidence roles. To avoid duplication, detailed outcome-specific extraction status is provided separately in Supplementary Table S1, which documents adult separability, postoperative CSF leak, meningitis/intracranial infection, neurological/vascular complications, postoperative seizures, and the reason each report contributed or did not contribute quantitatively. Supplementary Table S2 provides detailed source-verified characteristics of studies contributing quantitative or comparative evidence, including country/institution, study design, sample size and age composition, pathology, EEA terminology/subtype, adult separability, and source-level descriptions or definitions of the reported complications. Mixed-age cohorts are explicitly identified and not treated as adult-only cohorts when adult-specific outcome counts cannot be independently separated.

### 3.3. Risk of Bias Summary

The risk-of-bias framework and key methodological concerns are summarized in Table 2, while study-level assessments for reports contributing to extractable quantitative outcomes are presented in Table 3. No randomized controlled trials were identified in this review. The evidence base consisted predominantly of retrospective cohort studies, non-randomized comparative studies, and case series. Comparative studies were assessed using the ROBINS-I tool, whereas non-comparative studies contributing pooled outcome data were evaluated using the Joanna Briggs Institute (JBI) Critical Appraisal Checklist for Case Series. Overall, the principal sources of bias included confounding by indication, selection bias, variable complication definitions, incomplete follow-up, and selective reporting of outcomes. Studies that did not provide exact extractable numerators and denominators for predefined outcomes were retained in the screening and extraction status summary Table 1 but were not included in the study-level risk-of-bias assessment. As shown in Table 3, most quantitatively contributing studies were judged to have a moderate-to-serious risk of bias, reflecting the observational nature of the available evidence and the limited number of studies with extractable complication data.

**Table 2:** Risk of bias framework and preliminary concerns.

Evidence Group	Applicable Tool	Risk-of-Bias Concern
Comparative EEA vs. transcranial studies	ROBINS-I	Confounding factors included indication, tumor size/location, surgical era, selection of approach, institutional learning curve, missing outcome data, and selective reporting.
Noncomparative pathology-specific case series	JBI Critical Appraisal Checklist for Case Series	Consecutive enrollment, complete inclusion, standardized complication definitions, and completeness of follow-up were assessed when exact outcome data contributed to the synthesis.
Mixed EEA skull base complication series	JBI or ROBINS-I depending on design	High duplicate cohort risk and limited pathology-specific extractability.
CSF leak/meningitis-focused studies	JBI or ROBINS-I depending on design	Useful for contextual complication rates and reconstruction-related risk factors; pooled only when adult intradural tumor numerators and denominators were independently extractable.

JBI = Joanna Briggs Institute; ROBINS-I = Risk Of Bias In Non-randomized Studies—of Interventions.

**Table 3:** Study-level risk-of-bias assessment for studies contributing extractable quantitative data.

Study	Evidence Contribution	Tool	Key Risk-of-Bias Concerns	Overall Judgment
Goldschmidt et al., 2020 [9]	Postoperative seizure estimate within overall complication profile; EEA versus open comparative seizure estimate	ROBINS-I	Nonrandomized approach selection; potential confounding by indication, tumor size/location, preoperative seizure status, surgical era, and antiepileptic prophylaxis; outcome extractable.	Serious risk of bias
Koutourousiou et al., 2013 [14]	CSF leak sensitivity/contextual evidence (mixed-age cohort)	JBI case series	Non-comparative case series; mixed-age composition (47 adults, 17 children) created indirectness for adult-only inference, and an exact adult-specific CSF leak numerator was not independently extractable. Whole-cohort data were restricted to sensitivity and contextual analyses.	Moderate risk of bias
Gardner et al., 2008, craniopharyngioma [18]	Meningitis and neurovascular/neurological complication synthesis	JBI case series	Small non-comparative case series; rare-event estimates imprecise; complication ascertainment extractable, but denominator and outcome definitions were not uniformly defined across domains.	Moderate to serious risk of bias
Gardner et al., 2008, anterior cranial base meningioma [3]	CSF leak, meningitis and neurovascular/neurological complication synthesis	JBI case series	Small noncomparative case series from an earlier reconstruction era; perioperative management and reporting definitions may differ from contemporary practices.	Moderate to serious risk of bias
Yu et al., 2021 [39]	CSF leak, meningitis and neurovascular/neurological complication synthesis	JBI case series	Single-institution consecutive case series; exact complication counts are extractable; residual concerns about non-comparative design and rare-event precision.	Moderate risk of bias
Liu et al., 2023 [15]	CSF leak sensitivity/contextual evidence only	JBI case series/contextual evidence	Mixed-age case series (age range 7–61 years); adult-specific outcome counts were not separable. Whole cohort data were excluded from the strict adult-only primary analysis.	Serious risk of bias for main adult-only inference
Koutourousiou et al., 2014 [34]	CSF leak exact-count synthesis; descriptive meningitis, neurovascular, mortality, and seizure data	JBI case series	Large single-center pathology-specific series; exact counts were extractable, but the long recruitment interval and evolving reconstruction practice introduced temporal and institutional confounding.	Moderate risk of bias

**Figure 2:**
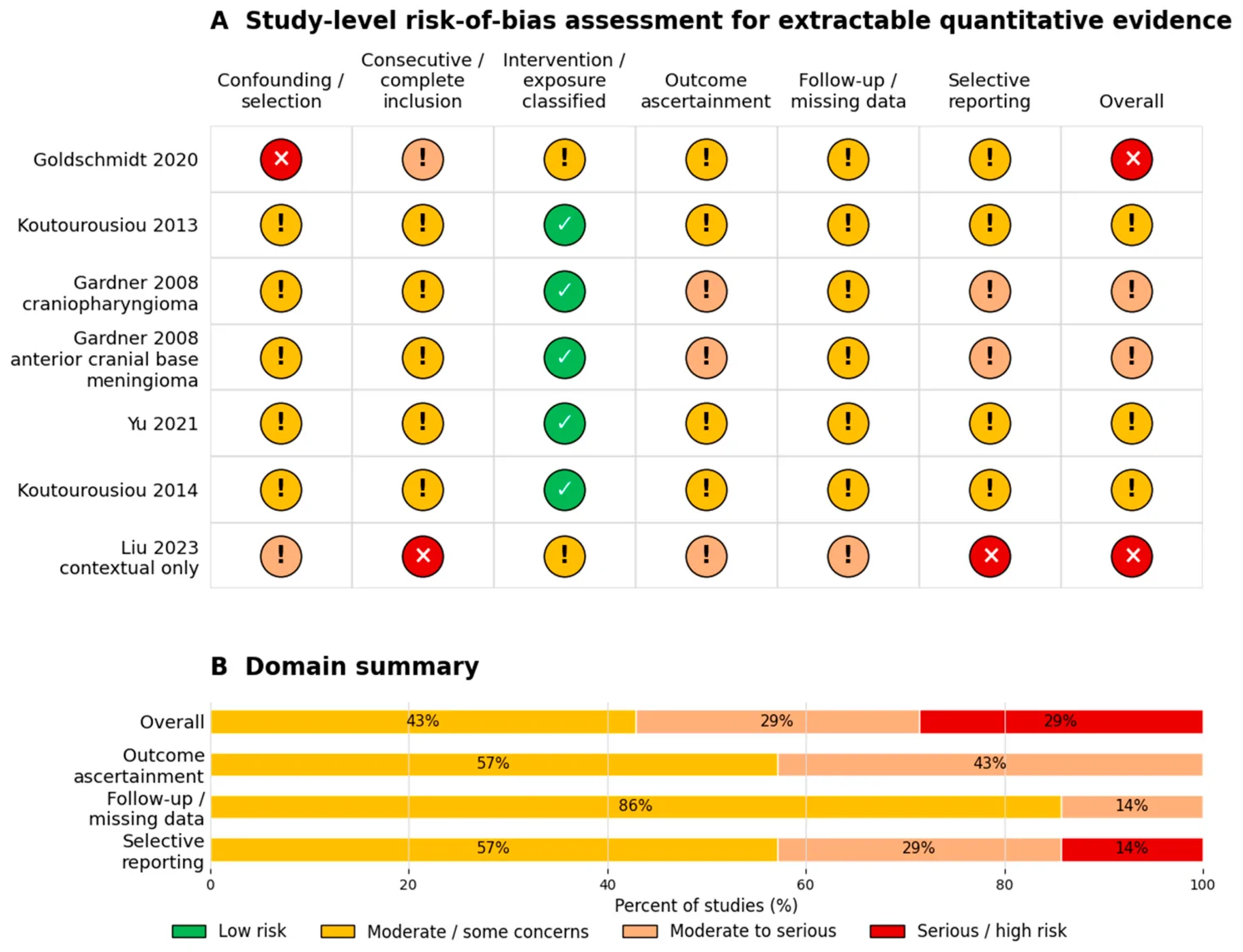
Traffic-light risk-of-bias assessment for studies contributing extractable quantitative data. Green indicates low risk, yellow indicates moderate/some concerns, orange indicates moderate-to-serious concerns, and red indicates serious/high risk of bias. Colored circles represent the study-level risk-of-bias judgment for the corresponding domain. The studies included are Goldschmidt et al. (2020) [9], Koutourousiou et al. (2013) [14], Gardner et al. (2008) [18], Gardner et al. (2008) [3], Yu et al. (2021) [39], Liu et al. (2023) [15], and Koutourousiou et al. (2014) [34]. Only studies with exact extractable outcome data are included.

Overall, the risk-of-bias framework was applied to studies contributing exact numerator/denominator data. The study-level and domain-specific risk-of-bias assessments are visually summarized in Figure 2, complementing the detailed study-level assessments presented in Table 3.

### 3.4. Postoperative Seizures Within the Overall Complication Profile

Postoperative seizures were evaluated as neurological complications within the broader safety profile. Goldschmidt et al. [9] reported four seizures among 577 EEA patients, and Koutourousiou et al. [34] reported no seizures among 75 patients with suprasellar meningioma. Across these two reports, 4/652 patients experienced postoperative seizures (0.6%; 95% exact CI, 0.2–1.6%). Goldschmidt et al. [9] reported 27 seizures among 481 open craniotomy patients and an odds ratio of 0.13 favoring EEA. Because only two reports provided verified adult intradural seizure counts and one report had zero events, a conventional pooled seizure meta-analysis was not performed. The seizure endpoint is presented as a crude descriptive total in Table 4 and interpreted as sparse evidence within the overall complication spectrum, rather than the dominant conclusion of this review.

### 3.5. Cerebrospinal Fluid Leak

Exact postoperative CSF leak counts suitable for the strict adult-only primary analysis were available in three reports: Gardner et al. [3] reported 14/35, Koutourousiou et al. [34] reported 19/75, and Yu et al. [39] reported 3/40 patients. Source-level verification showed that Koutourousiou et al. [14] included 47 adults and 17 children and reported 15/64 CSF leaks for the whole cohort, while Liu et al. [15] included patients within an age range of 7–61 years and reported 1/21. Because neither mixed-age report provided an independently extractable exact adult-specific CSF leak numerator, both were excluded from the strict adult-only primary synthesis and retained only for sensitivity/contextual analysis.

**Figure 3:**
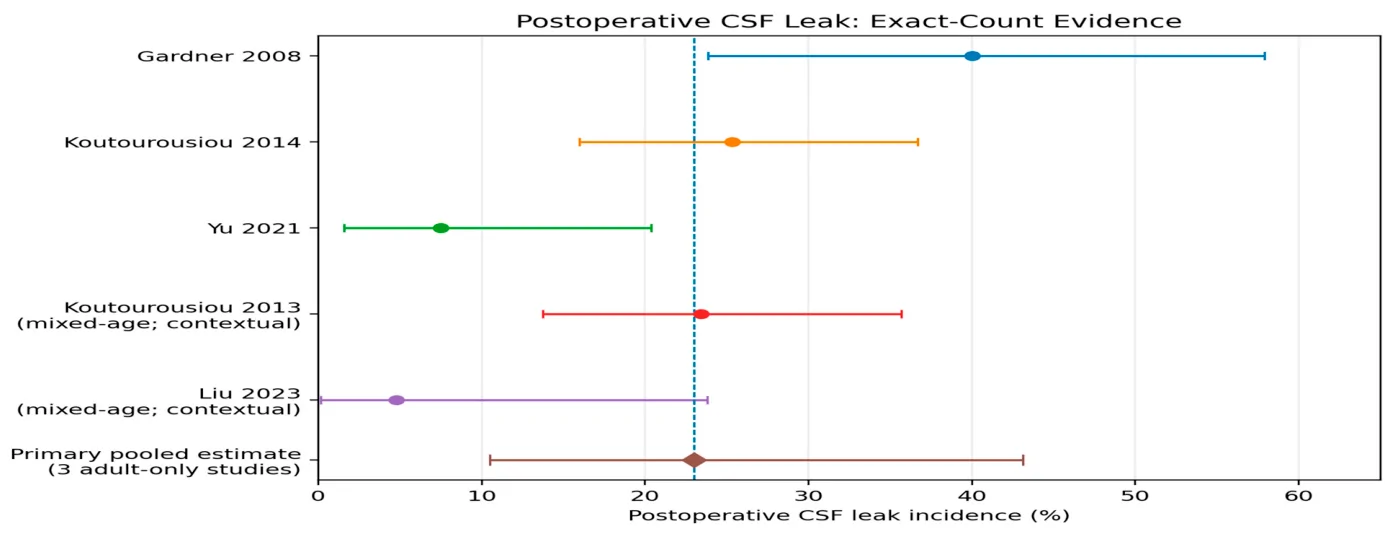
Forest plot of postoperative CSF-leak incidence across exact-count reports. The primary random-effects analysis includes three strictly adult-only studies: Gardner et al. (2008) [3], Koutourousiou et al. (2014) [34], and Yu et al. (2021) [39]. Koutourousiou et al. (2013) [14] and Liu et al. (2023) [15] are displayed as mixed-age contextual studies and are excluded from the strict adult-only primary pooled estimate. Points represent study-specific incidence estimates, and horizontal lines represent the corresponding 95% confidence intervals. The colored dashed vertical lines indicate the summary estimates from the strict adult-only primary analysis and the two progressively broader sensitivity/contextual analyses, as identified by the corresponding colors in the figure. The primary adult-only random-effects incidence was 23.0% (95% CI, 10.5–43.1%).

Using the three strictly adult-only exact-count reports, the primary random-effects CSF leak incidence was 23.0% (95% CI, 10.5–43.1%), with substantial heterogeneity (I^2^ = 78.7%). Adding the mixed-age Koutourousiou et al. [14] whole-cohort count yielded 23.8% (95% CI, 14.5–36.6%; I^2^ = 68.9%). A broader sensitivity/contextual analysis, including the mixed-age Liu et al. [15] report, yielded 21.1% (95% CI, 12.2–33.8%; I^2^ = 69.4%). These estimates are derived from a small, pathology- and center-heterogeneous evidence base and should not be interpreted as universal EEA complication rates. A formal funnel plot analysis or Egger’s test was not performed because only three studies contributed to the primary analysis. The study-specific CSF leak counts and incidences contributing to the strict adult-only primary synthesis are summarized in Table 5. The study-specific CSF-leak estimates and the strict adult-only random-effects pooled estimate are presented in Figure 3, with the two mixed-age reports displayed separately as contextual evidence.

### 3.6. Meningitis

Four reports provided exact meningitis counts: Gardner et al. [18] reported 0/16, Gardner et al. [3] reported 0/35, Koutourousiou et al. [34] reported 4/75, and Yu et al. [39] reported 2/40 cases.

**Figure 4:**
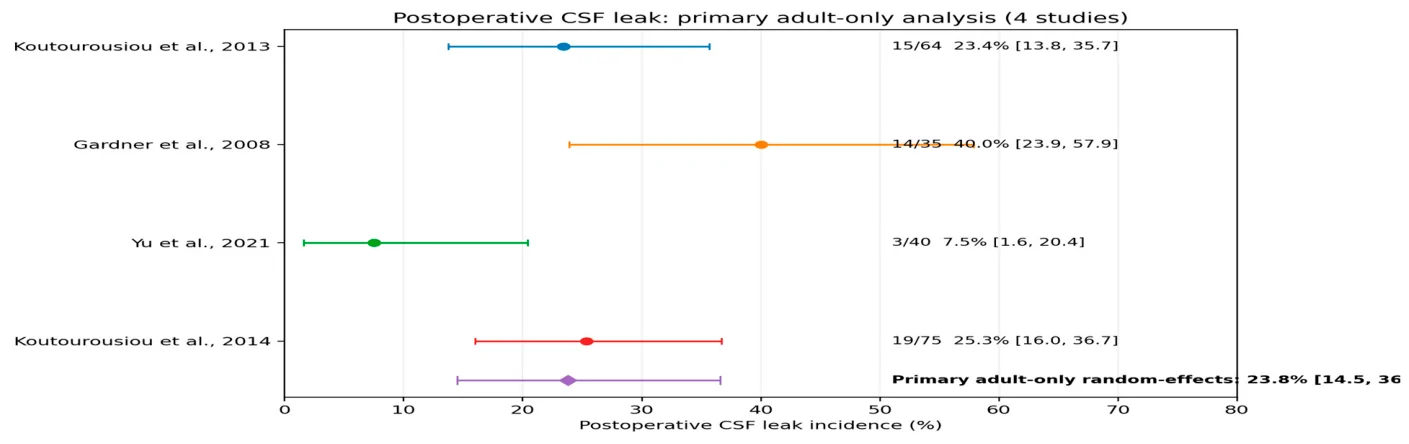
Study-specific meningitis estimates and crude descriptive total. Points represent the observed incidence for each individual study, and horizontal lines represent the corresponding 95% exact confidence intervals. Colors are used only to distinguish the individual studies and do not represent different analytical categories. The dashed vertical line indicates the crude descriptive incidence across the four contributing studies (6/166 patients; 3.6%) and is provided as a visual reference only; it does not represent a meta-analytic pooled estimate. The studies displayed are Gardner et al. (2008) [18], Gardner et al. (2008) [3], Yu et al. (2021) [39], Koutourousiou et al. (2013) [14], and Koutourousiou et al. (2014) [34].

Because two studies had zero events and the total number of events was small, meningitis was reported descriptively rather than being pooled. The crude incidence was 6/166 (3.6%; 95% exact confidence interval [CI], 1.3–7.7%). Study-specific meningitis estimates and the crude descriptive total are presented in Figure 4. This avoids a continuity-correction-driven estimate that would overstate the observed event proportions. The study-specific meningitis counts and crude incidence are presented in Table 6.

### 3.7. New Neurological Deficit, Vascular Injury or Stroke

Four reports provided exact data on new neurological deficits, vascular injury, or stroke-like complications: Gardner et al. [18] reported 1/16, Gardner et al. [3] reported 1/35, Koutourousiou et al. [34] reported 1/75, and Yu et al. [39] reported 1/40.

**Figure 5:**
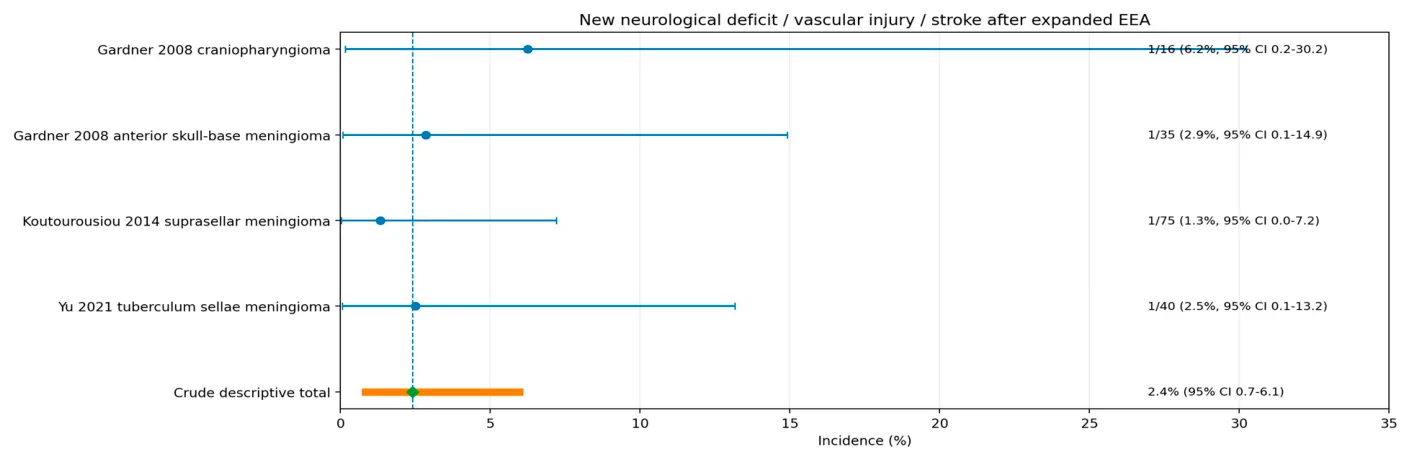
Study-specific neurological/vascular complication estimates and crude descriptive total. Points represent the observed incidence for each individual study, and horizontal lines represent the corresponding 95% exact confidence intervals. Colors are used only to distinguish the individual studies and do not represent different analytical categories. The dashed vertical line indicates the crude descriptive incidence across the four contributing studies (4/166 patients; 2.4%) and is provided as a visual reference only; it does not represent a meta-analytic pooled estimate. The studies displayed are Gardner et al. (2008) [18], Gardner et al. (2008) [3], Yu et al. (2021) [39], and Koutourousiou et al. (2014) [34]. The composite endpoint includes related but nonidentical neurological and vascular events.

Because the endpoint combined related but non-identical outcomes and only four events were observed, it was reported descriptively rather than being pooled. The crude incidence was 4/166 (2.4%; 95% exact CI, 0.7–6.1%). Study-specific neurological/vascular complication estimates and the crude descriptive total are presented in Figure 5. The study-specific neurological/vascular event counts and crude incidences are summarized in Table 7.

### 3.8. Comparative EEA Versus Transcranial Seizure Evidence

Goldschmidt et al. [9] provided the only directly extractable comparative seizure estimate. The study reported 4 postoperative seizures among 577 EEA patients and 27 among 481 open craniotomy patients (OR 0.13, 95% CI, 0.05–0.35). No pooled comparative analysis was performed; this remains single-study, nonrandomized evidence.

### 3.9. Subgroup and Sensitivity Analyses

Formal seizure subgroup analysis could not be performed because only two studies provided verified seizure counts. The CSF leak subgroup analysis by pathology remained underpowered and clinically heterogeneous. In the strict adult-only primary set, exact-count CSF leak rates ranged from 7.5% to 40.0%, and the random-effects incidence was 23.0%. Adding the mixed-age Koutourousiou et al., 2013 [14] cohort yielded 23.8%, while adding both mixed-age reports yielded 21.1%. These sensitivity analyses demonstrate that the principal interpretation remains constrained by sparse and heterogeneous evidence rather than by any single pooled value.

Studies were not excluded from this review merely because they were large or complication-focused studies. Instead, pathology-specific adult intradural counts were synthesized when separable, while mixed cohorts were summarized contextually to avoid combining pituitary, extradural, pediatric, or overlapping institutional data with the target population.

### 3.10. Exploratory Temporal and Era-Based Analysis

The publication-era comparison Table 8 is presented only as a descriptive record of observed reports and not as evidence of temporal improvement. Both the early and modern era groupings contain mixed-age contextual data, and the apparent differences are inseparable from pathology, center, reconstruction strategy, and the use of publication year rather than the operative year. Accordingly, the era display was excluded from the Abstract and Conclusion sections and was not used for causal inference.

**Figure 6:**
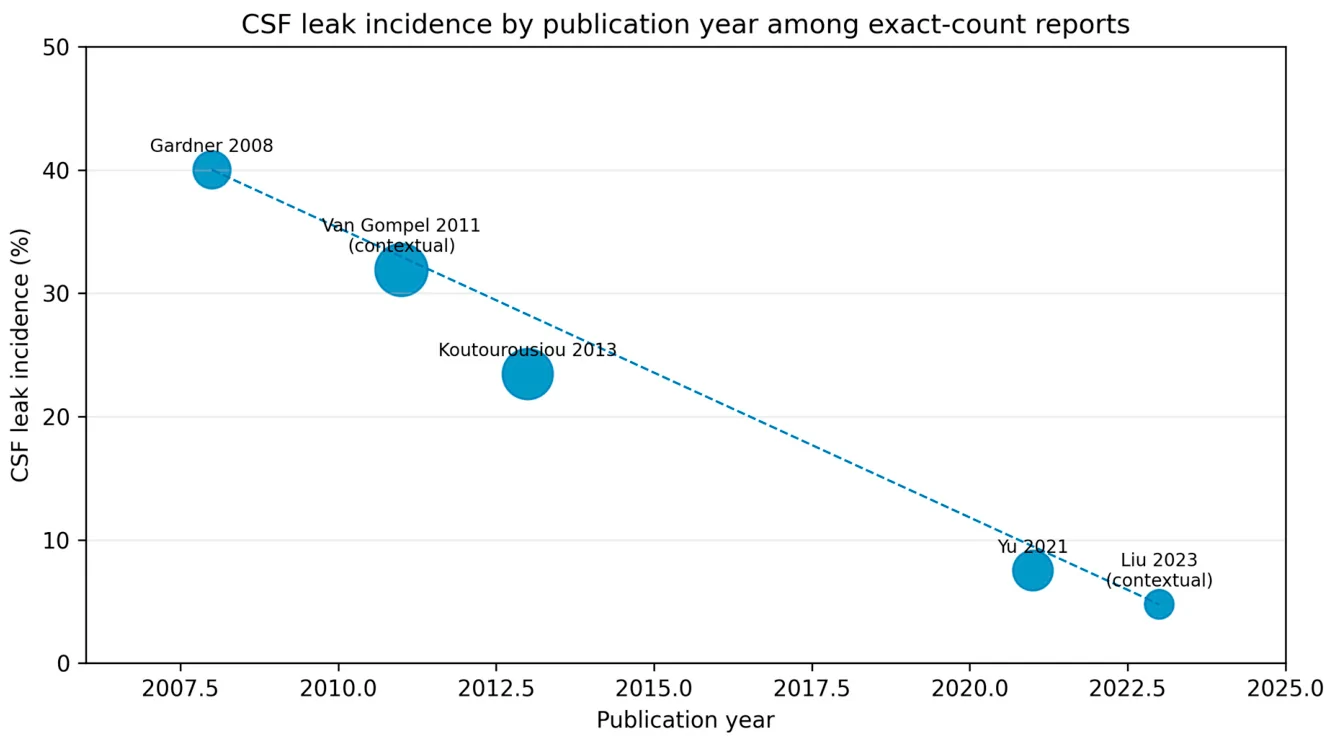
CSF leak incidence by publication year among exact-count reports. Each point represents the reported CSF-leak incidence from an individual report, plotted according to publication year. The dashed connecting line is included only to visually display the sequence of observed study-level estimates over time and does not represent a fitted regression, pooled estimate, or statistically tested temporal trend. Gardner et al. (2008) [18], Gardner et al. (2008) [3], Van Gompel et al. (2011) [32], Koutourousiou et al. (2013) [14], Yu et al. (2021) [39], Liu et al. (2023) [15] are shown only as a contextual early benchmark and were not included in pooled era estimates because of potential overlap with the primary series. Differences across publication years should not be interpreted causally because of heterogeneity in pathology, patient populations, reconstruction strategies, and center-level practice.

**Figure 7:**
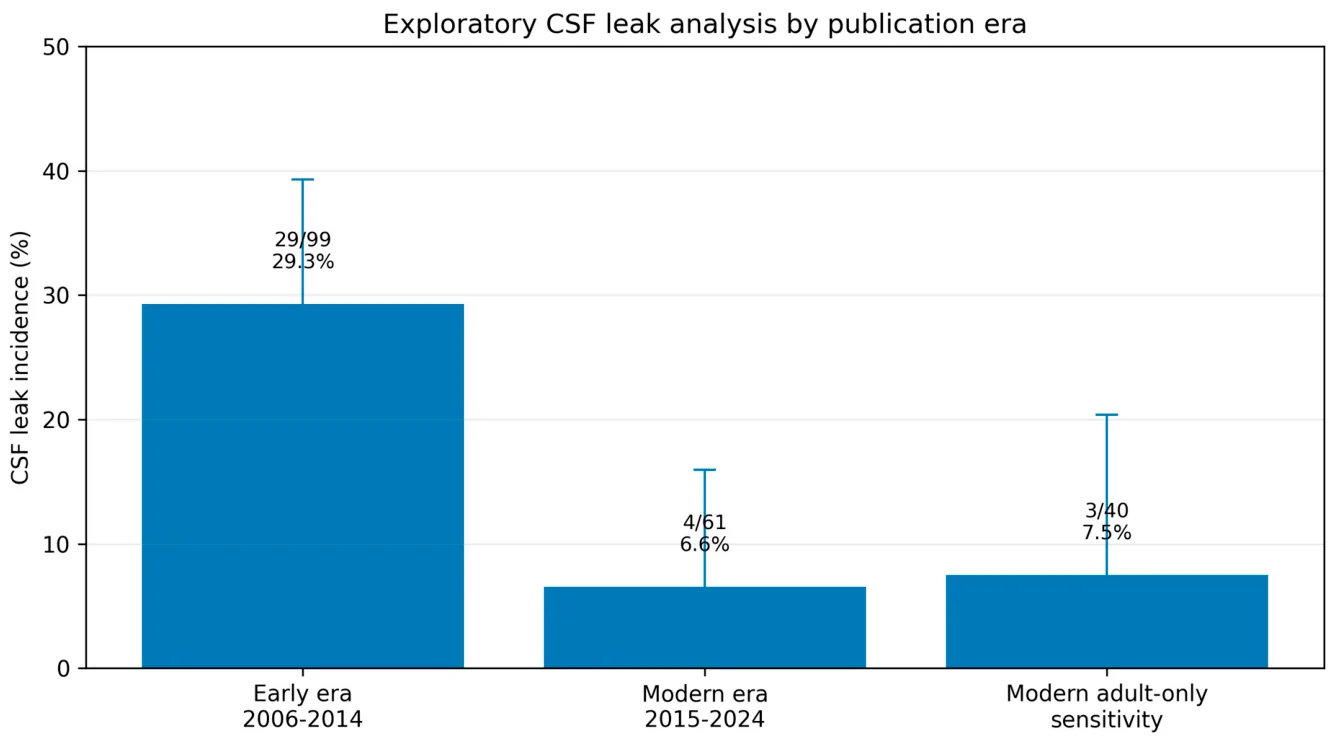
Non-inferential exploratory display of CSF-leak incidence by publication era. Bars represent the crude descriptive CSF-leak incidence for each displayed era/sensitivity group, and vertical error bars represent the corresponding 95% confidence intervals. The early-era (2006–2014) and modern-era (2015–2024) groups include mixed-age contextual evidence and therefore should not be interpreted as strictly adult-only estimates; the modern adult-only sensitivity estimate is based on a single adult-only report. No formal between-era statistical comparison or meta-regression was performed. The figure is descriptive and non-inferential; differences between groups should not be interpreted as evidence of temporal improvement because of differences in pathology, center-level practice, reconstruction strategies, age composition, and the use of publication year as a proxy for operative era.

Van Gompel et al. [32] remains an early contextual benchmark: the accompanying literature aggregate reported 22/69 CSF leaks, while the authors’ own 13-patient series reported none. Because the aggregate overlaps with the earlier primary series, it was not added to the quantitative denominator. The distribution of exact-count CSF-leak estimates by publication year is shown in Figure 6, with Van Gompel et al. retained only as a contextual early benchmark because of potential cohort overlap. An additional non-inferential exploratory display by publication era is provided in Figure 7; these temporal patterns should not be interpreted causally because publication year is only a proxy for operative era and is confounded by differences in pathology and center-level practice.

### 3.11. Summary of Findings

The principal findings are presented in Table 9. CSF leak was the only outcome suitable for random-effects pooling in a strictly adult-only pathology-specific exact-count subset, yielding 23.0% (95% CI, 10.5–43.1%; three studies; I^2^ = 78.7%). Adding the mixed-age Koutourousiou et al., 2013 [14] cohort yielded 23.8% (95% CI, 14.5–36.6%), and adding both mixed-age reports yielded 21.1% (95% CI, 12.2–33.8%). Meningitis occurred in 6/166 patients across four studies (3.6%), neurological/vascular complications in 4/166 across four studies (2.4%), and postoperative seizures in 4/652 across two studies (0.6%); these sparse outcomes were descriptive totals, not meta-analytic pooled estimates. The comparative seizure odds estimate was limited to a single non-randomized study. Large mixed EEA cohorts provided contextual rates but were not representative of adult intradural tumors. The principal large mixed EEA cohorts retained as contextual safety evidence, together with their reported complications and reasons for exclusion from quantitative pooling, are summarized in Table 10.

## 4. Discussion

This systematic review and meta-analysis evaluated the safety of intradural EEAs across a spectrum of complications. Among the 48 reports retained after full-text eligibility assessment and extraction-status assessment, CSF leak was suitable for quantitative synthesis in a small strictly adult-only exact-count subset, whereas meningitis, neurological/vascular events, and seizures were too sparse or heterogeneous for reliable pooled rare-event estimates and were therefore summarized descriptively. Large mixed EEA cohorts were retained as contextual evidence rather than omitted.

CSF leakage was the most frequently reported extractable complication. Across three strictly adult-only studies, 36 events occurred among 150 patients, with a random-effects incidence of 23.0% (95% CI, 10.5–43.1%; I^2^ = 78.7%). Adding the mixed-age Koutourousiou et al., 2013 [14] whole-cohort count yielded 23.8% (95% CI, 14.5–36.6%; I^2^ = 68.9%), and adding both mixed-age reports yielded 21.1% (95% CI, 12.2–33.8%; I^2^ = 69.4%). The wide study-level range, pathology differences, defect characteristics, reconstruction strategy, and center experience materially limit the generalizability of any pooled value. With only three primary studies, funnel plot asymmetry and Egger’s test were not considered reliable.

Meningitis was reported in 6/166 patients across four studies (3.6%; 95% exact CI, 1.3–7.7%). Neurological/vascular complications were reported in 4/166 patients across the same four studies (2.4%; 95% exact CI, 0.7–6.1%). These values are crude descriptive totals with Clopper–Pearson exact confidence intervals; no rare-event meta-analytic model and no continuity correction were used. Interpretation is additionally limited by non-identical source definitions, including differences in infection ascertainment and classification of transient/permanent neurological and vascular events.

The large complication datasets clarify why two-tier synthesis is necessary. Kassam et al. reported postoperative CSF leak in 15.9% of 800 mixed-age, mixed-pathology patients, Conger et al. reported 9 repair failures and 6 meningitis cases across 551 mixed parasellar operations, and Hardesty et al. reported 61 CSF leaks and 6 meningitis cases across 1002 procedures [55,58,59]. These cohorts are stronger for broad institutional safety surveillance, but their adult intradural tumor subsets are not separable and therefore cannot be pooled as if they represented the same target population.

The publication-era display should not be interpreted as evidence that CSF leaks have declined over time. Only a few pathology-mismatched, center-specific reports are available, and the publication year is an inadequate proxy for the operative era. Improvements in reconstruction remain biologically plausible, but this dataset cannot estimate their independent temporal effect [32,56,57,58,59].

In addition to advances in reconstruction, postoperative imaging may further improve the management of patients at risk of CSF leakage. Ajlan et al. demonstrated that the distribution of intracranial air on early postoperative computed tomography independently predicts postoperative CSF leak following endoscopic skull base surgery, suggesting that early imaging may facilitate identification of high-risk patients and allow for earlier intervention when appropriate [60].

Meningitis and neurological/vascular complications were less frequent in the extractable pathology-specific series but remained clinically important. The descriptive rates were 3.6% and 2.4%. The neurological/vascular endpoint combined new deficit, vascular injury, and infarction; so, it should not be interpreted as a uniform stroke rate [3,18,34,39].

The optimal strategy for preoperative nasal antiseptic preparation remains unclear. In the randomized three-arm trial by Ajlan et al., no clinically meaningful or statistically significant difference in postoperative infectious complications was observed among the preparation protocols; no individual protocol demonstrated superiority [61].

Although vascular injury was uncommon in the present review, it remains one of the most catastrophic complications of expanded endoscopic endonasal surgery because injury to the internal carotid artery may result in massive hemorrhage, ischemic stroke, pseudoaneurysm formation, or death. Contemporary reports emphasize meticulous anatomical orientation, rapid hemorrhage control, multidisciplinary management, and postoperative vascular surveillance following suspected injury [62]. Furthermore, intraoperative magnetic resonance imaging has recently been described as a valuable adjunct for confirming vascular integrity and assisting intraoperative decision-making following suspected internal carotid artery injury [63].

Postoperative seizures formed a narrower evidence stream within the broader complication analysis. Goldschmidt et al. [9] reported 4/577 events and lower seizure odds than open craniotomy, while Koutourousiou et al. [34] reported 0/75 events. The combined crude incidence was 0.6%; however, the comparative conclusion remains limited to one non-randomized cohort and is vulnerable to confounding by indication, tumor characteristics, and perioperative management.

Beyond minimizing major neurological morbidity, contemporary expanded endoscopic approaches increasingly emphasize the preservation of functional outcomes, particularly olfactory function, during anterior skull base surgery. Recent technical refinements, including the endoscopic L-shape approach for anterior skull base meningiomas, have demonstrated that carefully tailored surgical corridors may facilitate maximal safe resection while preserving olfactory function in appropriately selected patients [64].

The certainty of these findings differed substantially across the outcomes. CSF leakage has the most consistent quantitative evidence in the available literature, although confidence in the pooled estimate remains limited by the small number of studies, substantial heterogeneity, and predominantly observational designs. Evidence for meningitis, neurological/vascular complications, and postoperative seizures is less certain because of sparse events, inconsistent outcome definitions, and a small number of contributing studies. Accordingly, the low observed frequencies of these secondary complications should not be interpreted as evidence that their risks are negligible.

From a clinical standpoint, counseling should address the entire spectrum of complications. CSF leak and reconstruction-related morbidity are the most consistently reported signals, whereas meningitis and neurovascular events require attention despite sparse data. Seizure prophylaxis should not be driven by limited EEA evidence. Current brain tumor guidelines do not support routine prophylactic antiseizure medication in seizure-naïve patients; perioperative treatment should remain individualized [10,65,66]. Future studies should report standardized adult-only intradural outcomes, exact counts, reconstruction methods, and clearly defined neurological events.

A contextual regional series by Alsaleh et al., including the work of Ajlan et al., also supports the need to report vascular complications separately from general neurological morbidity [67]. In that 45-case expanded transnasal skull base experience, CSF leak was the most common complication (9/45; 20%), and major vascular injury occurred in 2/45 cases, including anterior cerebral/anterior communicating artery injury with infarction. Because that cohort included a mixed age range and broad pathology, it should not be pooled into the present adult intradural estimates, but it is directly relevant to the discussion of rare, high-impact ACA-related complications.

### Strengths and Limitations

This review realigned the objective, search, extraction, and interpretation of the full complication spectrum. It separated pathology-specific adult intradural evidence from large mixed EEA cohorts, added an outcome-specific supplementary extraction table, and avoided continuity correction-driven pooling of sparse rare events. The resulting estimates are more transparent; however, they remain limited by the underlying reporting quality.

This study has several limitations. A complete record-level search log was not available. Although the aggregate counts shown in the retained PRISMA diagram could be reported, a verifiable category-by-category breakdown of the reasons for all 32 full-text exclusions could not be reconstructed and retrospectively created without supporting documentation. Only three strictly adult-only reports provided exact CSF leak counts for the primary analysis, four provided meningitis and neurological/vascular counts, and two reported seizure counts. The small number of contributing studies, substantial heterogeneity for CSF leak, sparse events for secondary outcomes, and predominantly observational single-center designs limit the precision and generalizability of the results. The small number of studies contributing to the primary meta-analysis also precluded a reliable formal assessment of publication bias using funnel plot asymmetry or Egger’s regression. Two otherwise relevant CSF leak reports were mixed-age and lacked independently extractable exact adult-specific CSF leak numerators; therefore, their whole-cohort results were restricted to sensitivity/contextual analyses. Complication definitions and ascertainment methods, follow-up duration, reconstruction techniques, and age separability varied across reports, introducing additional clinical and methodological heterogeneities. A strict sensitivity analysis was not feasible because standardized definitions were not consistently available for all variables. Potential institutional overlap among large mixed cohorts further limits the synthesis. Structured certainty judgments are qualitative rather than formal GRADE ratings and should be interpreted accordingly.

## 5. Conclusions

CSF leak remains the most consistently reported postoperative complication following expanded EEAs for adult intradural skull base tumors. In the strict adult-only primary analysis, the random-effects incidence was 23.0% (95% CI, 10.5–43.1%); however, this estimate was derived from only three studies and was accompanied by substantial heterogeneity, limiting its precision and generalizability of the results. Meningitis, neurological/vascular complications, and postoperative seizures were uncommon in the available extractable data, and, because of sparse events and heterogeneous reporting, were more appropriately summarized descriptively than meta-analyzed. Large mixed EEA cohorts provide useful contextual information regarding institutional safety but should not be considered interchangeable with evidence specific to adult intradural pathology. Future studies should prioritize standardized complication definitions, adult-separable reporting, exact outcome counts, and detailed reporting of reconstruction techniques to improve the reliability and clinical applicability of the complication estimates.

## Figures and Tables

**Table 1:** Overview of the 48 reports retained after full-text eligibility assessment.

No.	Study	Tumor/Pathology Group	Evidence Role
1	Goldschmidt et al., 2020 [9]	Mixed intradural skull base tumors	Quantitative/descriptive endpoint evidence
2	Frank G et al., 2006 [16]	Craniopharyngioma	Retained extraction-status evidence; not quantitatively usable
3	de Divitiis E et al., 2007 [17]	Craniopharyngioma	Retained extraction-status evidence; not quantitatively usable
4	Gardner PA et al., 2008 [18]	Craniopharyngioma	Quantitative/descriptive endpoint evidence
5	Stamm AC et al., 2008 [19]	Craniopharyngioma	Retained extraction-status evidence; not quantitatively usable
6	Cavallo LM et al., 2009 [20]	Recurrent/residual craniopharyngioma	Retained extraction-status evidence; not quantitatively usable
7	Jane JA Jr et al., 2010 [21]	Adult craniopharyngioma	Retained extraction-status evidence; not quantitatively usable
8	Koutourousiou M et al., 2013 [14]	Craniopharyngioma	Sensitivity/contextual evidence
9	Cavallo LM et al., 2014 [22]	Craniopharyngioma	Retained extraction-status evidence; not quantitatively usable
10	Wannemuehler TJ et al., 2016 [23]	Adult craniopharyngioma; EEA vs. transcranial	Retained extraction-status evidence; not quantitatively usable
11	Moussazadeh N et al., 2016 [24]	Craniopharyngioma; EEA vs. transcranial	Retained extraction-status evidence; not quantitatively usable
12	Jeswani S et al., 2016 [25]	Craniopharyngioma and related tumors	Retained extraction-status evidence; not quantitatively usable
13	Park HR et al., 2017 [26]	Craniopharyngioma	Retained extraction-status evidence; not quantitatively usable
14	Dho YS et al., 2018 [27]	Craniopharyngioma	Retained extraction-status evidence; not quantitatively usable
15	Feng Z et al., 2022 [28]	Recurrent craniopharyngioma	Retained extraction-status evidence; not quantitatively usable
16	Nie C et al., 2022 [29]	Craniopharyngioma; transcranial vs. EEA	Retained extraction-status evidence; not quantitatively usable
17	Liu J et al., 2023 [15]	Craniopharyngioma	Sensitivity/contextual evidence
18	Kohli G et al., 2024 [30]	Recurrent/residual craniopharyngioma	Retained extraction-status evidence; not quantitatively usable
19	de Divitiis E et al., 2007 [31]	Tuberculum sellae meningioma	Retained extraction-status evidence; not quantitatively usable
20	Gardner PA et al., 2008 [3]	Anterior cranial base meningioma	Quantitative/descriptive endpoint evidence
21	Van Gompel JJ et al., 2011 [32]	Anterior fossa meningioma	Sensitivity/contextual evidence
22	Khan OH et al., 2014 [33]	Olfactory groove/tuberculum sellae meningioma	Retained extraction-status evidence; not quantitatively usable
23	Koutourousiou M et al., 2014 [34]	Suprasellar meningioma	Quantitative/descriptive endpoint evidence
24	Koutourousiou M et al., 2014 [35]	Olfactory groove meningioma	Retained extraction-status evidence; not quantitatively usable
25	Bander ED et al., 2018 [36]	Tuberculum/planum meningioma; EEA vs. transcranial	Retained extraction-status evidence; not quantitatively usable
26	Elshazly K et al., 2018 [37]	Tuberculum sellae meningioma	Retained extraction-status evidence; not quantitatively usable
27	Magill ST et al., 2018 [38]	Tuberculum sellae meningioma; transcranial vs. transsphenoidal	Retained extraction-status evidence; not quantitatively usable
28	Yu P et al., 2021 [39]	Tuberculum sellae meningioma	Quantitative/descriptive endpoint evidence
29	Algattas HN et al., 2021 [40]	Anterior cranial fossa meningioma	Retained extraction-status evidence; not quantitatively usable
30	Frank G et al., 2006 [41]	Chordoma/chondrosarcoma	Retained extraction-status evidence; not quantitatively usable
31	Carrabba G et al., 2008 [42]	Clival lesions	Retained extraction-status evidence; not quantitatively usable
32	Dehdashti AR et al., 2008 [43]	Clival chordoma	Retained extraction-status evidence; not quantitatively usable
33	Zhang Q, 2008 [44]	Clival chordoma/chondrosarcoma	Retained extraction-status evidence; not quantitatively usable
34	Stippler M et al., 2009 [45]	Clival chordoma	Retained extraction-status evidence; not quantitatively usable
35	Fraser JF et al., 2010 [46]	Clival lesions	Retained extraction-status evidence; not quantitatively usable
36	Holzmann D et al., 2010 [47]	Clivus chordoma	Retained extraction-status evidence; not quantitatively usable
37	Vaz-Guimaraes F et al., 2019 [48]	Epidermoid/dermoid cysts	Retained extraction-status evidence; not quantitatively usable
38	Madhok R et al., 2010 [49]	Rathke cleft cyst	Retained extraction-status evidence; not quantitatively usable
39	Laufer I et al., 2007 [50]	Mixed suprasellar lesions	Retained extraction-status evidence; not quantitatively usable
40	Ceylan S et al., 2009 [51]	Mixed midline skull base lesions	Retained extraction-status evidence; not quantitatively usable
41	Dehdashti AR et al., 2009 [52]	Anterior cranial base/suprasellar lesions	Retained extraction-status evidence; not quantitatively usable
42	Fatemi N et al., 2009 [53]	Craniopharyngioma and tuberculum sellae meningioma	Retained extraction-status evidence; not quantitatively usable
43	Kurschel S et al., 2011 [54]	Non-adenomatous sellar and skull base lesions	Retained extraction-status evidence; not quantitatively usable
44	Kassam AB et al., 2011 [55]	Mixed EEA skull base complications	Sensitivity/contextual evidence
45	Ivan ME et al., 2015 [56]	CSF leak/meningitis risk factors	Sensitivity/contextual evidence
46	Fraser S et al., 2018 [57]	CSF leak risk factors	Sensitivity/contextual evidence
47	Conger A et al., 2019 [58]	Repair failure/meningitis	Sensitivity/contextual evidence
48	Hardesty DA et al., 2022 [59]	EEA complications	Sensitivity/contextual evidence

The evidence role provides only a concise overview. The detailed outcome-specific extraction status and reasons for non-use are provided in Supplementary Table S1.

**Table 4:** Summary of the quantitative synthesis and comparative evidence.

Endpoint	Evidence Base	Estimate	Heterogeneity	Model/Interpretation
Postoperative seizure	Goldschmidt et al., 2020 [9]; Koutourousiou et al., 2014 [34]	4/652; 0.6% crude incidence (95% exact CI, 0.2–1.6%)	Not estimated	Descriptive two-study evidence
CSF leak (primary strict adult-only)	3 adult-only exact-count reports [3,34,39]	23.0%, 95% CI, 10.5–43.1%	I^2^ = 78.7%	Logit inverse-variance random-effects; DerSimonian–Laird
CSF leak (sensitivity/contextual 1)	4 exact-count reports including mixed-age Koutourousiou et al. [14]	23.8%, 95% CI, 14.5–36.6%	I^2^ = 68.9%	Adds Koutourousiou 2013 [14] whole-cohort data; not the primary adult-only estimate
CSF leak (sensitivity/contextual 2)	5 exact-count reports including mixed-age Koutourousiou et al. [14] and Liu et al. [15]	21.1%, 95% CI, 12.2–33.8%	I^2^ = 69.4%	Broader mixed-age sensitivity/contextual analysis; not the primary adult-only estimate
Meningitis	4 reports [3,18,34,39]	6/166; 3.6% crude incidence (95% exact CI, 1.3–7.7%)	Not estimated	Descriptive; no continuity correction
New neurological deficit/vascular injury/stroke	4 reports [3,18,34,39]	4/166; 2.4% crude incidence (95% exact CI, 0.7–6.1%)	Not estimated	Descriptive composite endpoint; no continuity correction
EEAs vs. open seizure odds	Goldschmidt et al., 2020 [9]	OR 0.13, 95% CI, 0.05–0.35	Not pooled	Comparative single-study estimate

CI: confidence interval; CSF: cerebrospinal fluid; EEA: expanded endoscopic endonasal approach; OR: odds ratio.

**Table 5:** CSF leak exact count synthesis.

Study	Events/Patients	Incidence
Gardner et al., 2008, anterior cranial base meningioma [3]	14/35	40.0%
Koutourousiou et al., 2014 [34]	19/75	25.3%
Yu et al., 2021 [39]	3/40	7.5%
Koutourousiou et al., 2013 [14]	15/64	23.4%; mixed-age sensitivity/contextual evidence
Liu et al., 2023 [15]	1/21	4.8%; mixed-age sensitivity/contextual evidence
Primary strict adult-only random-effects estimate	36/150	23.0%, 95% CI, 10.5–43.1%
Sensitivity/contextual estimate 1 (adds Koutourousiou 2013) [14]	51/214	23.8%, 95% CI, 14.5–36.6%
Sensitivity/contextual estimate 2 (adds Koutourousiou 2013 [14] and Liu 2023 [15])	52/235	21.1%, 95% CI, 12.2–33.8%

CSF: cerebrospinal fluid.

**Table 6:** Meningitis: exact-count descriptive synthesis.

Study	Events/Patients	Incidence
Gardner et al., 2008, craniopharyngioma [18]	0/16	0%
Gardner et al., 2008, anterior cranial base meningioma [3]	0/35	0%
Yu et al., 2021 [39]	2/40	5.0%
Koutourousiou et al., 2014 [34]	4/75	5.3%
Crude descriptive total	6/166	3.6%, 95% exact CI, 1.3–7.7%

**Table 7:** New neurological deficit/vascular injury/stroke exact count descriptive synthesis.

Study	Events/Patients	Incidence
Gardner et al., 2008, craniopharyngioma [18]	1/16	6.3%
Gardner et al., 2008, anterior cranial base meningioma [3]	1/35	2.9%
Yu et al., 2021 [39]	1/40	2.5%
Koutourousiou et al., 2014 [34]	1/75	1.3%
Crude descriptive total	4/166	2.4%, 95% exact CI, 0.7–6.1%

**Table 8:** Exploratory CSF leak analysis by publication era.

Era/Sensitivity Set	Included Reports	Events/Patients	Incidence, 95% CI	Interpretation
Early era (2006–2014)	Gardner et al., 2008 [3]; Koutourousiou et al., 2013 [14]	29/99	29.3% (20.6–39.3%)	Descriptive only; includes the mixed-age Koutourousiou 2013 [14] cohort and therefore is not an adult-only era estimate.
Modern era (2015–2024)	Yu et al., 2021 [39]; Liu et al., 2023 [15]	4/61	6.6% (1.8–15.9%)	Descriptive only; includes the mixed-age Liu 2023 [15] cohort and therefore is not an adult-only era estimate.
Modern strict adult-only subset	Yu et al., 2021 [39]	3/40	7.5% (1.6–20.4%)	Single adult-only report; no inference about temporal changes is possible.
Van Gompel contextual benchmark	Van Gompel et al., 2011 [32]	22/69	31.9% (21.2–44.2%)	Discussed narratively only because of the risk of overlap with the early primary series.

**Table 9:** Summary of findings and structured qualitative certainty judgments for endpoints with extractable data.

Endpoint	No. of Studies	Participants/Events	Effect Estimate	Structured Qualitative Certainty Judgment/Limitations
Postoperative seizure	2	4/652	0.6% crude incidence (95% exact CI, 0.2–1.6%)	Very low confidence: only two reports and four events; descriptive estimates only.
CSF leak	3 primary strict adult-only studies	36/150	23.0%, 95% CI, 10.5–43.1%	Low confidence: observational evidence, only three strictly adult-only studies, substantial heterogeneity (I^2^ = 78.7%), and a wide confidence interval; mixed-age reports were excluded from the primary analysis.
Meningitis	4	6/166	3.6% crude incidence (95% exact CI, 1.3–7.7%)	Very low confidence: observational evidence, sparse events, zero-event studies, imprecision, and variable definitions; descriptive estimates only.
New neurological deficit/vascular injury/stroke	4	4/166	2.4% crude incidence (95% exact CI, 0.7–6.1%)	Very low confidence: observational evidence, only four events, imprecision, non-identical composite definitions, and descriptive estimates only.
EEA vs. open seizure odds	1 comparative cohort	EEA 4/577; open 27/481	OR 0.13, 95% CI, 0.05–0.35	Very low confidence: a single nonrandomized comparative study with a serious risk of confounding; no pooled comparative estimate.

CI = confidence interval; EEA = expanded endoscopic endonasal approach; OR = odds ratio.

**Table 10:** Large mixed EEA cohorts retained as contextual safety evidence are shown alongside the strict adult-only pathology-specific CSF leak synthesis.

Evidence Source	Cohort Size/Population	Reported Complications	Interpretation/Reason Not Pooled
Strict adult-only pathology-specific primary synthesis	3 studies; 150 patients; strictly adult-only, pathology-specific exact-count evidence	Postoperative CSF leak: 36/150; random-effects incidence 23.0% (95% CI, 10.5–43.1%; I^2^ = 78.7%)	Primary adult-only quantitative synthesis was performed. This is shown as a reference for contextual comparison only; the mixed EEA cohorts below are not directly comparable with this estimate.
Kassam et al., 2011 [55]	800 patients; mixed age (3–96 years), mixed-pathology EEA cohort	CSF leak 15.9%; intracranial infection 13/800; transient neurological deficit 20/800; permanent neurological deficit 14/800; mortality 7/800	Contextual institutional safety evidence only is available. Adult intradural tumor subgroup was not separable.
Conger et al., 2019 [58]	509 patients; 551 operations; mixed parasellar cohort; pituitary adenomas 66%	CSF repair failure 9/551 operations; meningitis 6/551 operations	Contextual institutional safety evidence only is available. Predominantly pituitary/mixed procedures; adult intradural tumor subgroup not separable.
Hardesty et al., 2022 [59]	1002 procedures; large mixed-pathology EEA cohort	CSF leak 61/1002 (6.1%); meningitis 6/1002	Contextual institutional safety evidence only is available. Procedure-level mixed pathology; adult intradural tumor subgroup not separable.
Ivan et al., 2015 [56]	98 patients; expanded EEA complication/risk-factor cohort	CSF leak and CNS infection risk factors reported	Contextual risk factor evidence only. Exact adult intradural tumor counts were not separable.
Fraser et al., 2018 [57]	Contextual intradural EEA CSF-leak risk-factor cohort	Reconstruction-related CSF leak risk-factor evidence	Contextual risk factor evidence only. Potential institutional overlap and no independent pathology-specific counts for the prespecified synthesis.

[Abbreviations: CI, confidence interval; CNS, central nervous system; CSF, cerebrospinal fluid; EEA, expanded endoscopic endonasal approach]. Note: Large mixed cohorts are informative for institutional safety surveillance and reconstruction-related context but were not combined with the adult intradural pathology-specific estimate because the target subgroup was not independently extractable. Their reported CSF leak rates should not be interpreted as directly comparable pooled estimates.

## Data Availability

All aggregate data extracted for the present synthesis are reported in the manuscript tables and figures. Additional extraction forms, search logs, and analytic codes should be made available by the corresponding author upon reasonable request, where permitted.

## Supplementary Materials

The following supporting information can be downloaded at: https://www.mdpi.com/article/10.3390/brainsci16080887/s1, Supplementary Table S1, Outcome-specific extraction status of all 48 retained reports; Supplementary Table S2, Source-verified characteristics of studies contributing quantitative or comparative evidence; Supplementary Table S3: PRISMA_2020_checklist.
