# Supplementary material for: Complication Spectrum Following Expanded Endoscopic Endonasal Surgery for Intradural Skull Base Tumors: A Systematic Review and Meta-Analysis
Source: Brain Sci. 2026 Aug 20;16(8):887. doi: 10.3390/brainsci16080887 (PMC13511667; doi:10.3390/brainsci16080887)
Supplement: Supplementary file 1 [file brainsci-16-00887-s001.zip › brainsci-4486027-supplementary.pdf]

Supplementary Table S1. Outcome-specific extraction status of all 48 retained reports

No.	Study	Pathology / population	Adult separability	Postoperative CSF leak	Meningitis / intracranial infection	Neurological / vascular complication	Postoperative seizure	Quantitative use / reason not used
1	Goldschmidt, et al. 2020[9]	Mixed intradural skull base tumors	EEA intradural cohort used for seizure endpoint	NR for prespecified quantitative synthesis	NR for prespecified quantitative synthesis	NR for prespecified quantitative synthesis	EEA 4/577; open comparator 27/481	Quantitative comparative/descriptive seizure evidence was collected.
2	Frank G, et al. 2006[14]	Craniopharyngioma	Adult separability/outcome denominator insufficient for quantitative use in retained extraction	NR / exact adult intradural count not extractable	NR / exact adult intradural count not extractable	NR / exact adult intradural count not extractable	NR / exact adult intradural count not extractable	Not quantitatively usable for the prespecified outcomes.
3	de Divitiis E, et al. 2007[15]	Craniopharyngioma	Adult separability/outcome denominator insufficient for quantitative use in retained extraction	NR / exact adult intradural count not extractable	NR / exact adult intradural count not extractable	NR / exact adult intradural count not extractable	NR / exact adult intradural count not extractable	Not quantitatively usable for the prespecified outcomes.
4	Gardner PA, et al. 2008[16]	Craniopharyngioma	16-patient eligible contribution in retained extraction	Reported as percentage; exact event count not pooled	0/16 bacterial meningitis	1/16 vascular injury/new neurological deficit	NR	Used descriptively for meningitis and neurological/vascular outcomes; CSF leak was not pooled.
5	Stamm AC, et al. 2008[17]	Craniopharyngioma	Adult separability/outcome denominator insufficient for quantitative use in retained extraction	NR / exact adult intradural count not extractable	NR / exact adult intradural count not extractable	NR / exact adult intradural count not extractable	NR / exact adult intradural count not extractable	Not quantitatively usable for the prespecified outcomes.
6	Cavallo LM, et al. 2009[18]	Recurrent/residual craniopharyngioma	Adult separability/outcome denominator insufficient for quantitative use in retained extraction	NR / exact adult intradural count not extractable	NR / exact adult intradural count not extractable	NR / exact adult intradural count not extractable	NR / exact adult intradural count not extractable	Not quantitatively usable for the prespecified outcomes.
7	Jane JA Jr, et al. 2010[19]	Adult craniopharyngioma	Adult separability/outcome denominator insufficient for quantitative use in retained extraction	NR / exact adult intradural count not extractable	NR / exact adult intradural count not extractable	NR / exact adult intradural count not extractable	NR / exact adult intradural count not extractable	Not quantitatively usable for the prespecified outcomes.
8	Koutourousiou M, et al. 2013[20]	Craniopharyngioma	Mixed age: 47 adults, 17 children; adult-specific CSF event numerator not separable	15/64 overall cohort	NR / not adult-separable	NR / not adult-separable	NR / not adult-separable	Contextual/sensitivity only; excluded from strict adult-only primary CSF leak analysis.
9	Cavallo LM, et al. 2014[21]	Craniopharyngioma	Adult separability/outcome denominator insufficient for quantitative use in retained extraction	NR / exact adult intradural count not extractable	NR / exact adult intradural count not extractable	NR / exact adult intradural count not extractable	NR / exact adult intradural count not extractable	Not quantitatively usable for the prespecified outcomes.
10	Wannemuehler TJ, et al. 2016[22]	Adult craniopharyngioma; EEA vs transcranial	Adult separability/outcome denominator insufficient for quantitative use in retained extraction	NR / exact adult intradural count not extractable	NR / exact adult intradural count not extractable	NR / exact adult intradural count not extractable	NR / exact adult intradural count not extractable	Not quantitatively usable for the prespecified outcomes.
11	Moussazadeh N, et al. 2016[23]	Craniopharyngioma; EEA vs transcranial	Adult separability/outcome denominator insufficient for quantitative use in retained extraction	NR / exact adult intradural count not extractable	NR / exact adult intradural count not extractable	NR / exact adult intradural count not extractable	NR / exact adult intradural count not extractable	Not quantitatively usable for the prespecified outcomes.
12	Jeswani S, et al. 2016[24]	Craniopharyngioma and related tumors	Adult separability/outcome denominator insufficient for quantitative use in retained extraction	NR / exact adult intradural count not extractable	NR / exact adult intradural count not extractable	NR / exact adult intradural count not extractable	NR / exact adult intradural count not extractable	Not quantitatively usable for the prespecified outcomes.
13	Park HR, et al. 2017[25]	Craniopharyngioma	Adult separability/outcome denominator insufficient for quantitative use in retained extraction	NR / exact adult intradural count not extractable	NR / exact adult intradural count not extractable	NR / exact adult intradural count not extractable	NR / exact adult intradural count not extractable	Not quantitatively usable for the prespecified outcomes.

No.	Study	Pathology / population	Adult separability	Postoperative CSF leak	Meningitis / intracranial infection	Neurological / vascular complication	Postoperative seizure	Quantitative use / reason not used
14	Dho YS, et al. 2018[26]	Craniopharyngioma	Adult separability/outcome denominator insufficient for quantitative use in retained extraction	NR / exact adult intradural count not extractable	NR / exact adult intradural count not extractable	NR / exact adult intradural count not extractable	NR / exact adult intradural count not extractable	Not quantitatively usable for the prespecified outcomes.
15	Feng Z, et al. 2022[27]	Recurrent craniopharyngioma	Adult separability/outcome denominator insufficient for quantitative use in retained extraction	NR / exact adult intradural count not extractable	NR / exact adult intradural count not extractable	NR / exact adult intradural count not extractable	NR / exact adult intradural count not extractable	Not quantitatively usable for the prespecified outcomes.
16	Nie C, et al. 2022[28]	Craniopharyngioma; transcranial vs EEA	Adult separability/outcome denominator insufficient for quantitative use in retained extraction	NR / exact adult intradural count not extractable	NR / exact adult intradural count not extractable	NR / exact adult intradural count not extractable	NR / exact adult intradural count not extractable	Not quantitatively usable for the prespecified outcomes.
17	Liu J, et al. 2023[29]	Craniopharyngioma	Mixed age (7–61 years); adult-specific outcome counts not separable	1/21 overall cohort	NR / not adult-separable	NR / not adult-separable	NR / not adult-separable	Contextual/sensitivity only; excluded from strict adult-only primary CSF leak analysis.
18	Kohli G, et al. 2024[30]	Recurrent/residual craniopharyngioma	Adult separability/outcome denominator insufficient for quantitative use in retained extraction	NR / exact adult intradural count not extractable	NR / exact adult intradural count not extractable	NR / exact adult intradural count not extractable	NR / exact adult intradural count not extractable	Not quantitatively usable for the prespecified outcomes.
19	de Divitiis E, et al. 2008[31]	Tuberculum sellae meningioma	Adult separability/outcome denominator insufficient for quantitative use in retained extraction	NR / exact adult intradural count not extractable	NR / exact adult intradural count not extractable	NR / exact adult intradural count not extractable	NR / exact adult intradural count not extractable	Not quantitatively usable for the prespecified outcomes.
20	Gardner PA, et al. 2008[3]	Anterior cranial base meningioma	Adult-separable/eligible EEA cohort in retained extraction	14/35	0/35 bacterial meningitis	1/35 new neurological deficit	NR	It was used in strict adult-only CSF leak analysis and descriptive secondary outcomes.
21	Van Gompel JJ, et al. 2011[32]	Anterior fossa meningioma	Own series reported separately; literature aggregate overlaps primary evidence	Own series 0/13; accompanying literature meta-analysis 22/69	NR	NR	NR	Contextual only; literature aggregate not pooled because of overlap in results.
22	Khan OH, et al. 2014[33]	Olfactory groove/tuberculum sellae meningioma	Adult separability/outcome denominator insufficient for quantitative use in retained extraction	NR / exact adult intradural count not extractable	NR / exact adult intradural count not extractable	NR / exact adult intradural count not extractable	NR / exact adult intradural count not extractable	Not quantitatively usable for the prespecified outcomes.
23	Koutourousiou M, et al. 2014[34]	Suprasellar meningioma	75 adults; age range 36–88 years	19/75	4/75	1/75 vascular injury/new neurological deficit	0/75	It was used in strict adult-only CSF leak analysis and descriptive secondary outcomes.
24	Koutourousiou M, et al. 2014[35]	Olfactory groove meningioma	Adult separability/outcome denominator insufficient for quantitative use in retained extraction	NR / exact adult intradural count not extractable	NR / exact adult intradural count not extractable	NR / exact adult intradural count not extractable	NR / exact adult intradural count not extractable	Not quantitatively usable for the prespecified outcomes.
25	Bander ED, et al. 2018[36]	Tuberculum/planum meningioma; EEA vs transcranial	Adult separability/outcome denominator insufficient for quantitative use in retained extraction	NR / exact adult intradural count not extractable	NR / exact adult intradural count not extractable	NR / exact adult intradural count not extractable	NR / exact adult intradural count not extractable	Not quantitatively usable for the prespecified outcomes.
26	Elshazly K, et al. 2018[37]	Tuberculum sellae meningioma	Adult separability/outcome denominator insufficient for quantitative use in retained extraction	NR / exact adult intradural count not extractable	NR / exact adult intradural count not extractable	NR / exact adult intradural count not extractable	NR / exact adult intradural count not extractable	Not quantitatively usable for the prespecified outcomes.

No.	Study	Pathology / population	Adult separability	Postoperative CSF leak	Meningitis / intracranial infection	Neurological / vascular complication	Postoperative seizure	Quantitative use / reason not used
27	Magill ST, et al. 2018[38]	Tuberculum sellae meningioma; transcranial vs transsphenoidal	Adult separability/outcome denominator insufficient for quantitative use in retained extraction	NR / exact adult intradural count not extractable	NR / exact adult intradural count not extractable	NR / exact adult intradural count not extractable	NR / exact adult intradural count not extractable	Not quantitatively usable for the prespecified outcomes.
28	Yu P, et al. 2021[39]	Tuberculum sellae meningioma	Eligible 40-patient cohort in retained extraction	3/40	2/40 (following CSF leakage)	1/40 A2/ACA-branch bleeding with postoperative infarction	0/40	It was used in strict adult-only CSF leak analysis and descriptive secondary outcomes.
29	Algattas HN, et al. 2021[40]	Anterior cranial fossa meningioma	Adult separability/outcome denominator insufficient for quantitative use in retained extraction	NR / exact adult intradural count not extractable	NR / exact adult intradural count not extractable	NR / exact adult intradural count not extractable	NR / exact adult intradural count not extractable	Not quantitatively usable for the prespecified outcomes.
30	Frank G, et al. 2006[41]	Chordoma/chondrosarcoma	Adult separability/outcome denominator insufficient for quantitative use in retained extraction	NR / exact adult intradural count not extractable	NR / exact adult intradural count not extractable	NR / exact adult intradural count not extractable	NR / exact adult intradural count not extractable	Not quantitatively usable for the prespecified outcomes.
31	Carrabba G, et al. 2008[42]	Clival lesions	Adult separability/outcome denominator insufficient for quantitative use in retained extraction	NR / exact adult intradural count not extractable	NR / exact adult intradural count not extractable	NR / exact adult intradural count not extractable	NR / exact adult intradural count not extractable	Not quantitatively usable for the prespecified outcomes.
32	Dehdashti AR, et al. 2008[43]	Clival chordoma	Adult separability/outcome denominator insufficient for quantitative use in retained extraction	NR / exact adult intradural count not extractable	NR / exact adult intradural count not extractable	NR / exact adult intradural count not extractable	NR / exact adult intradural count not extractable	Not quantitatively usable for the prespecified outcomes.
33	Zhang Q, 2008[44]	Clival chordoma/chondrosarcoma	Adult separability/outcome denominator insufficient for quantitative use in retained extraction	NR / exact adult intradural count not extractable	NR / exact adult intradural count not extractable	NR / exact adult intradural count not extractable	NR / exact adult intradural count not extractable	Not quantitatively usable for the prespecified outcomes.
34	Stippler M, et al. 2009[45]	Clival chordoma	Adult separability/outcome denominator insufficient for quantitative use in retained extraction	NR / exact adult intradural count not extractable	NR / exact adult intradural count not extractable	NR / exact adult intradural count not extractable	NR / exact adult intradural count not extractable	Not quantitatively usable for the prespecified outcomes.
35	Fraser JF, et al. 2010[46]	Clival lesions	Adult separability/outcome denominator insufficient for quantitative use in retained extraction	NR / exact adult intradural count not extractable	NR / exact adult intradural count not extractable	NR / exact adult intradural count not extractable	NR / exact adult intradural count not extractable	Not quantitatively usable for the prespecified outcomes.
36	Holzmann D, et al. 2010[47]	Clivus chordoma	Adult separability/outcome denominator insufficient for quantitative use in retained extraction	NR / exact adult intradural count not extractable	NR / exact adult intradural count not extractable	NR / exact adult intradural count not extractable	NR / exact adult intradural count not extractable	Not quantitatively usable for the prespecified outcomes.
37	Vaz-Guimaraes F, et al. 2019[48]	Epidermoid/dermoid cysts	Adult separability/outcome denominator insufficient for quantitative use in retained extraction	NR / exact adult intradural count not extractable	NR / exact adult intradural count not extractable	NR / exact adult intradural count not extractable	NR / exact adult intradural count not extractable	Not quantitatively usable for the prespecified outcomes.
38	Madhok R, et al. 2010[49]	Rathke cleft cyst	Adult separability/outcome denominator insufficient for quantitative use in retained extraction	NR / exact adult intradural count not extractable	NR / exact adult intradural count not extractable	NR / exact adult intradural count not extractable	NR / exact adult intradural count not extractable	Not quantitatively usable for the prespecified outcomes.
39	Laufer I, et al. 2007[50]	Mixed suprasellar lesions	Adult separability/outcome denominator insufficient for quantitative use in retained extraction	NR / exact adult intradural count not extractable	NR / exact adult intradural count not extractable	NR / exact adult intradural count not extractable	NR / exact adult intradural count not extractable	Not quantitatively usable for the prespecified outcomes.
40	Ceylan S, et al. 2009[51]	Mixed midline skull base lesions	Adult separability/outcome denominator insufficient for quantitative use in retained extraction	NR / exact adult intradural count not extractable	NR / exact adult intradural count not extractable	NR / exact adult intradural count not extractable	NR / exact adult intradural count not extractable	Not quantitatively usable for the prespecified outcomes.

No.	Study	Pathology / population	Adult separability	Postoperative CSF leak	Meningitis / intracranial infection	Neurological / vascular complication	Postoperative seizure	Quantitative use / reason not used
41	Dehdashti AR, et al. 2009[52]	Anterior cranial base/suprasellar lesions	Adult separability/outcome denominator insufficient for quantitative use in retained extraction	NR / exact adult intradural count not extractable	NR / exact adult intradural count not extractable	NR / exact adult intradural count not extractable	NR / exact adult intradural count not extractable	Not quantitatively usable for the prespecified outcomes.
42	Fatemi N, et al. 2009[53]	Craniopharyngioma and tuberculum sellae meningioma	Adult separability/outcome denominator insufficient for quantitative use in retained extraction	NR / exact adult intradural count not extractable	NR / exact adult intradural count not extractable	NR / exact adult intradural count not extractable	NR / exact adult intradural count not extractable	Not quantitatively usable for the prespecified outcomes.
43	Kurschel S, et al. 2011[54]	Non-adenomatous sellar and skull base lesions	Adult separability/outcome denominator insufficient for quantitative use in retained extraction	NR / exact adult intradural count not extractable	NR / exact adult intradural count not extractable	NR / exact adult intradural count not extractable	NR / exact adult intradural count not extractable	Not quantitatively usable for the prespecified outcomes.
44	Kassam AB, et al. 2011[55]	Mixed EEA skull base complications	Mixed age 3–96 years; mixed pathology; target adult intradural subgroup not separable	15.9% (exact event count not used)	13/800 intracranial infection	20/800 transient neurological deficit; 14/800 permanent neurological deficit	NR	Contextual institutional safety evidence only is available.
45	Ivan ME, et al. 2015[56]	CSF leak/meningitis risk factors	Mixed/combined procedures; target adult intradural tumor subgroup not separable	Reported; exact target-subgroup count not separable	CNS infection risk factors reported; exact target-subgroup count not separable	NR	NR	Contextual risk factor evidence only.
46	Fraser S, et al. 2018[57]	CSF leak risk factors	Potential institutional overlap; independent pathology-specific target counts unavailable	Reported as intradural EEA risk-factor evidence; independent pathology-specific count not used	NR	NR	NR	Contextual risk factor evidence only.
47	Conger A, et al. 2019[58]	Repair failure/meningitis	Mixed parasellar cohort; pituitary adenomas 66%; target subgroup not separable	Repair failure 9/551 operations	6/551 operations	NR	NR	Contextual institutional safety evidence only is available.
48	Hardesty DA, et al. 2022[59]	EEA complications	Procedure-level mixed pathology; target adult intradural subgroup not separable	61/1002 procedures	6/1002 procedures	NR	NR	Contextual institutional safety evidence only is available.

[Abbreviations. CSF, cerebrospinal fluid; EEA, expanded endoscopic endonasal approach; ACA, anterior cerebral artery; NR, exact outcome information not reported or not independently extractable for the eligible adult intradural subgroup from the retained extraction.]

'Contextual' evidence was retained for sensitivity, comparative, risk-factor, or institutional safety interpretation but was not treated as interchangeable with the strict adult intradural primary synthesis. Koutourousiou et al. (2013) and Liu et al. (2023) were mixed-age cohorts without independently separable exact adult CSF-leak event numerators and were therefore excluded from the strict adult-only primary CSF-leak analysis.

Table S2. Source-verified characteristics of studies contributing quantitative or comparative evidence

Study	Country / institution	Design	Sample / adult separability	Pathology	EEA subtype / source terminology	Complication definition / source-level description
Gardner et al., 2008[16] - craniopharyngioma	USA; Department of Neurological Surgery, University of Pittsburgh Medical Center, Pittsburgh, Pennsylvania	Retrospective case series	16 patients; retained as an adult-eligible cohort in the review	Suprasellar craniopharyngioma	Endoscopic expanded endonasal resection / expanded endonasal approach	Bacterial meningitis: 0/16; vascular injury resulting in a new neurological deficit: 1/16. Source terminology was retained, and no post-hoc standardized definition was imposed.
Gardner et al., 2008[3] - anterior cranial base meningioma	USA; University of Pittsburgh School of Medicine, Pittsburgh, Pennsylvania	Clinical case series	35 EEA patients; adult-eligible cohort in the retained review	Anterior cranial base meningioma	Endoscopic endonasal resection / expanded endonasal approach	Postoperative CSF leak 14/35, bacterial meningitis 0/35, new neurological deficit 1/35 after postoperative hemorrhage, and mortality 0/35.
Koutourousiou et al., 2013[20]	USA; University of Pittsburgh, Pittsburgh, Pennsylvania	Retrospective case series	64 total: 47 adults and 17 children; mixed-age cohort	Craniopharyngioma	Endoscopic endonasal surgery (EES)	Overall postoperative CSF leak 15/64 (23.4%). The source reported CSF leak rates of 27.7% in adults and 11.8% in children, but an exact adult event numerator was not independently extractable; therefore, the whole-cohort count was retained only as sensitivity/contextual evidence.

Study	Country / institution	Design	Sample / adult separability	Pathology	EEA subtype / source terminology	Complication definition / source-level description
Koutourousiou et al., 2014[34]	USA; University of Pittsburgh Medical Center, Pittsburgh, Pennsylvania	Retrospective review / case series	75 adults; reported age range 36-88 years	Suprasellar meningioma, predominantly tuberculum sellae / planum sphenoidale	Endoscopic endonasal surgery (EES)	Postoperative CSF leak 19/75; meningitis 4/75 associated with postoperative CSF leaks; artery of Heubner injury resulting in neurological deficit 1/75; postoperative seizures 0/75; and one death within 1 month.
Yu et al., 2021[39]	China; Department of Neurosurgery, First Affiliated Hospital of China Medical University, Shenyang, Liaoning	Consecutive institutional case series	40 patients; reported age range 42-73 years (adult-only)	Tuberculum sellae meningioma	Expanded endoscopic endonasal approach (EEEA)	Postoperative CSF leakage occurred in 3/40 patients, meningitis following CSF leakage in 2/40, A2/ACA-branch bleeding followed by postoperative acute cerebral infarction in 1/40, and seizures in 0/40.
Liu et al., 2023[29]	China; Department of Neurosurgery, Chongqing General Hospital, Chongqing	Case series	21 patients; reported age range 7-61 years; mixed-age cohort	Craniopharyngioma	Endoscopic endonasal transsphenoidal approach	Postoperative CSF leak 1/21 (4.8%). The source also reported two transient seizures and two transient infections. Adult-specific outcome counts were not separable; therefore, whole-cohort outcomes were retained only as contextual/sensitivity evidence.
Goldschmidt et al., 2020[9]	USA; principally University of Pittsburgh Medical Center / University of Pittsburgh School of Medicine	Retrospective comparative cohort	577 EEA patients; open comparator also reported	Mixed intradural skull base tumors	Expanded endoscopic endonasal approach (EEA) versus open approach	Postoperative seizure endpoint: 4/577 patients with EEA. Seizure ascertainment was not based on a standardized routine EEG screening.

EEA: expanded endoscopic endonasal approach; EEEA: expanded endoscopic endonasal approach; EES: endoscopic endonasal surgery; CSF: cerebrospinal fluid; ACA: anterior cerebral artery. Complication terminology reflects the definitions or clinical descriptions reported in the original publications, rather than imposing post hoc standardized definitions. Koutourousiou et al. (2013) included 47 adults and 17 pediatric patients, and Liu et al. (2023) included patients aged 7-61 years; Because exact adult-specific CSF leak numerators were not independently extractable, their whole-cohort CSF leak counts were excluded from the strict adult-only primary meta-analysis and retained only in sensitivity/contextual analyses.
